# The Health of the Dentist—Means of Preserving

**Published:** 1869-03

**Authors:** H. M’Cullum


### THE HEALTH OF THE DENTIST—MEANS OF PRESERVING.

BY H. M'CULLUM, D. D. S.

The following article was called into existence in behalf of the Dental profession exclusively, and the writer engaged in the labors incident to its production with this distinct object in view; but, as the work progressed, the nature of the subject and the irresistible logic of circumstances led to a departure from the original design, so far as any attempt to present the general laws of hygiene, on which the physical welfare of the entire human family is dependent. Still, as the munificent liberality of some of the prominent members of the profession led to its production, and zeal for the honor and advancement of the profession inspired and sustained the writer during the period of its conception and gestation, or incubation, it is deemed but reasonable that it should first be submitted to the consideration and acceptance of the class or profession in whose behalf and through whose influence it was brought into existence, and if the effort meet the approbation of those for whom it was more especially prepared, and if such a step be thought advisable, it may ultimately be presented to the public at large through the medium of the profession.

At first sight it might appear unnecessary to discuss the general principles on which the health of mankind is founded, in order to meet expectations; but, on second thought, I arrive at the conclusion that as Dentists are but mortal men like every body else, and subject to the same physical and pathological laws, under the control of the same vital forces, it would be the proper course to endeavor to make a special application of the general laws of hygiene, to the special conditions in which Dental practitioners are generally found.

The thought might very naturally present itself here, that no one is competent to practice as a Dentist until he possesses a sufficient knowledge of the conditions of health to enable him to successfully guard his own interests in this direction, and if any one should presumptiously thrust himself into such a position without this primary, this indispensable qualification, he could not well sicken and die much too soon for his own good and the benefit of all concerned.

This view of the case would be altogether just and appropriate, but for the existence of one indisputable, all pervading and most humiliating fact, viz: that all human efforts and human attainments are imperfect and incomplete. None are so correct but they exhibit some deviation, none so full and perfect but there is something wanting; hence, it becomes us to cast the mantle of charity over the frailties and errors of our fellow-men, and endeavor to supply each other's lack of knowledge as we have ability and opportunity.

We seldom acquire knowledge, practical, useful knowledge, except in the direction and within the scope of our efforts. It is too frequently the case that the single aim and hope of the Dental student is not so much to gain an understanding of the physical organism and vital forces, such as shall enable him to establish and maintain a harmonious co-operation throughout the entire system, but rather that they may acquire a sufficient knowledge of special anatomy and handicraft skill, and, perhaps, social polish as shall command the approval of his compeers and reach the pockets of his patrons, and hence, notwithstanding the text-books that propose to set forth the laws of physical organization and vital action, in all their wonderful and charmingly beautiful mysteries, are placed within his reach, and every incentive to urge him to improve the auspicious moments, is brought to bear (except, indeed, the hope of immediate pecuniary gain) to induce him to improve the passing opportunity in laying up a fund of general knowledge that shall illuminate his pathway through all future existence; yet, notwithstanding all this, numbers of young men pass through a preparatory course of training, and come out at the end experts in repairing decayed teeth and supplying substitutes for such as are lost; but beyond this the treasures of wisdom and knowledge have been opened to them comparatively in vain.

And such is human nature, or human frailty, we can only see one thing distinctly at a time. If we attempt to see more our sense of perception becomes confused and chaotic; our effort proves a failure. Thus, not only Dentists, but all departments of society are left to assume the grave and responsible duties of life with a most deplorable deficiency in the direction of a proper knowledge of the conditions on which life and health are secured to mankind.

Oppressed with an overwhelming sense of my own inadequacy, I address myself to the task, only promising to do the best I can, under the circumstances, to place this matter in as clear a light as I shall find practicable.

In more respects than one man may be regarded as a triple individuality, holding three separate and distinct relations; first, to himself as an individual; second, to his fellow-men as a member of society; third, to his Creator as a subordinate dependent creature. His existence throughout its whole course is dependent on these vital conditions or functions, an interruption or suspension of the activity of either of which must instantly prove fatal. The harmonious co-operation of all these together comprehends the perfection of physical health; an excess or deficiency in any one of these functions is disease or derangement of health; interruption or suspension is death. I regard it quite unnecessary, other than to keep up the connection, to state that these vital functions are nutrition, respiration and circulation; that each has its separate and distinct system of organs and field of operations.

Next in order may be named the osseous, muscular and nervous systems, each with their own peculiar organization, and exclusive and appropriate functions.

Then the spiritual, sentient and physical, or material elements of his being; his moral, social and individual relations, complex and complicated beyond computation or comprehension.

Before we are prepared to engage in any enterprise, with a reasonable prospect of success, we should first understand the object at which we aim, fully and clearly; next, that we have at our command the means with which to conduct the operation; and, also, that we have confidence in our own ability to prosecute the enterprise to full success. It does not comport with the plan of this paper to exhibit in all their diversified details the complex conditions that underlie the health of mankind in all the various circumstances and occupations of life. We only propose to attempt to trace out some of the general laws of nature, that affect the sanitary condition of our race, and perhaps, endeavor to make a special application of these laws to the condition of such as are engaged in the practice of Dentistry. A large part of the details of the knowledge of hygienic laws must be gathered by the wayside of life. Like all other useful knowledge, it is too cumbrous, or too etherial, or too something, to carry a supply from the outset of life, even if we had the necessary outfit at starting, and, besides, it sometimes becomes stale if kept too long.

The outline of anatomy and physiology should be taught early in life—as early as the mind is capable of grasping such subjects ; they should be made a part of common school education, in preference to some of the subjects that are now taught at an untold expense of time and labor, in the morning of life, when every moment spent in idleness or misdirected effort, tends to blight the whole future course of existence.

Another error that men too frequently fall into is, that when they grow up to be five and a half or six feet high (instead of arriving at years of discretion) divide their time and attention between the pursuit of some sensual gratification, and an effort to secure the means wherewith to gratify their selfish or sensual desires, or in the pursuit of wealth under the sole impulse of covetous desires, which is another manifestation of selfishness of a low grade.

"But," some one may inquire, "what has all this to do with the health of the members of the Dental profession?" Perhaps nothing as Dentists exclusively or separately considered, but Dentists are only men, and as such are liable to all the infirmities and limitations that pertain to humanity, and no one can reasonably hope to succeed in the pursuit of health or any other object of value or interest, who ignores his identification with our common humanity, and in the further prosecution of this subject I shall frequently refer to the landmarks of human nature, as I understand them, for guidance and assurance that we are in the right way.

One of the first duties that devolves on us as Dentists is to gain and maintain a full and correct understanding of our own peculiarities, our mental and physical capabilities, our sanitary condition, our hereditary bias towards health or disease, our power of reaction or constitutional elasticity. This is necessary, in order to enable us to put forth the necessary efforts to secure and preserve our own health. If we are prone to any marked deviation from a condition of full and correct health, it is of the first importance that we understand the difficulty from the beginning. If we find ourselves laboring under any serious difficulty or derangement of health, it is altogether imprudent and impolitic to engage in such a vocation as the practice of Dentistry. If we fall a victim to disease while in the practice, our first step should be to abandon our professional labors, or, at least, suspend them until our health is restored. It is a duty that we owe to ourselves that we never suffer our health to be lost for want of due care on our part—that we always keep our line of communication with a healthy basis, open and unobstructed. It is a duty we owe to our patrons that we at all times hold ourselves prepared to afford them the services of a sound and healthy mind and body. No one with diseased lungs, or suffering from those prolonged chronic or nervous affections that frequently prove to be so tedious and troublesome, is justified in assuming the duties of a Dentist until his own health is restored. Besides the liability to aggravate his own case, and thereby lessen the prospects of recovery, there is an appearance of immodesty in proffering to assume the care of the health of others, while his own is deranged or impaired. No intelligent patient, no individual of good sense and correct taste would be pleased to be brought in contact with a subject of tedious and painful disease in that way; no one of correct judgment would choose to submit the care of their teeth or any of the appendages or appurtenances of their oral cavity to the care of a sick man. Besides, the tendency that most diseases have to emigrate from one individual to another, when brought in contact or close proximity, it would be altogether unreasonable to expect to find an invalid in a mental, nervous or muscular condition, such as would properly qualify him for the performance of the grave and responsible duties of a Dentist. But this is not quite to the purpose, which I understand to be to so advise those already in the practice of Dentistry as to enable them to secure and maintain such a state of health as shall qualify them for the successful performance of the duties of their important and responsible vocation, rather than to select candidates for the profession according to sanitary condition, &c.

The first condition of health that claims attention is, that the nutritive system be regularly supplied with a sufficient amount of appropriate food. This may be regarded as the most simple and most universally prevalent want among all organized beings ; yet so positive and imperative are its requirements that existence can be sustained (or rather sustain itself) but for a very brief time without it. In obedience, therefore, to this requisition, it is necessary that those who desire good health take due care to partake regularly and temperately of a portion of food, selected with proper regard to quality, quantity and adaptation to age, occupation, climate and constitutional peculiarity of the recipient; that the food be good in its kind, sound meat, fully matured and so prepared as not to change its chemical combinations. Remember that of the food we eat is constructed this curious fabric, which is so fearfully and wonderfully made, and that no architectural skill can make a safe and durable building of soft bricks and rotten timber. Not only is the general system weakended by improper diet, but the nutritive system rapidly becomes weakened or diseased. A large proportion of all the diseases prevalent in our day and our country arise and assail us from this quarter.

The next point that claims our attention is respiration. This is one of the vital functions, constantly and unremittingly active—not quite so liable to be misused as the appetite for food, still the doctrine of total depravity is fully sustained by the condition in which the lungs of large numbers of our fellow-men are formed. The immediate act or process of respiration is but partially under the control of the will, and is conducted regularly and correctly without the active control or attention of the will, as during sleep, or while the attention is fully occupied with other matters. The material demanded for respiration is the atmosphere ; though every where abundant and generally pure, it readily combines with deleterious and destructive gases, whenever and wherever brought in contact with them. When shut off from communication with the great atmospheric reservoir, it soon becomes stale and unfit for respiration. An admixture of the gases, resulting from combustion or the chemical decomposition of animal or vegetable substances, depreciates the quality or renders it unfit for the purposes of respiration, in proportion to the amount and character of the poisonous exhalation with which it is combined.

The third fundamental organic or animal function is circulation. This is carried on entirely independent of the control of the will, and, consequently, can only be reached by intermediate or secondary means. Its movements may be accelerated by the activity of the mental emotions, or by the introduction of nervous stimulants into the circulation by way of the stomach; by vigorous muscular exercise; in a word, all the activities of life stimulate the heart's action, which is but another word for increased activity of the circulation. It does not fall within the range of an article like this to describe minutely the structure or functions of different organs composing the human system; neither would it be necessary to refer to them in this general way, except for the purpose of laying a foundation for the remarks that we propose to make in reference to the origin of the derangement that arises, first in the functions of these organs, and ultimately in change of structure of the organs themselves, which is usually understood or designated by the term "disease."

In the prosecution of every important enterprise it is of the first importance that we frequently and intelligently examine the situation; that we understand distinctly what we propose to do, the means with which we have to operate, and the motives which prompt us to the undertaking, and not only at the outset, but during the prosecution, it is important that we maintain an intelligent oversight over every step till the labor is accomplished, and then sum up the re sult, together with expense of risk, toil, &c., incurred. As men, as organized animals, it is necessary that we take our food regularly, that it be good in its kind, adapted to our age, occupation, locality, &c. As Dentists, we are more frequently brought in contact with our patrons and friends in a way to interfere with our regular hours of diet, than persons in almost any other occupation. Now, for persons who, like some of the lower animals, do nothing else but eat, it would not make so much difference.

But the active Dentist has occupation sufficient to fill the hands of the most vigorous mental and executive capabilities. If the nervous energy be divided between manual or mental activity and digestive function, there is, of necessity, embarrassment, confusion and imperfect performance of both offices and rapid exhaustion of the vital energy. There is doubtless economical propriety in not only allowing sufficient time to chew and swallow our food in a thorough, deliberate and decorous manner, but in giving an hour after each meal to digestion, so that function shall not be impaired or weakened by a diversion of the nervous energy in a differerent direction. Though the digestion is not under the direct control of the will, it is one of those departments of vital economy that may be thwarted or impeded in its operation by willing the nerve force in an opposite direction. The Dentist does about the worst possible thing in this respect, who takes his food at odd times, as opportunity may serve or caprice may dictate; better omit dinner altogether than eat at twelve, one, two or three o'clock, just as he may chance to find leisure, and then return in eager haste to his task. There is no inconvenience in subsisting on two meals per day, other than that arising from a nonconformity with popular custom, and even one properly prepared and appropriated at regular intervals or periods, would be safer, better, and, in all respects, preferable to three or four taken at loose ends or random chances. It requires no elaborate argument to substantiate these facts in the mind of an intelligent observer, and to such only is this paper addressed. As it respects the respiration of Dentists, there is some special danger to be guarded against from this quärter. In the first place, the operating-room can hardly be sufficiently ventilated, even if the pent up atmosphere of the plethoric and smoky cities could furnish the needed article of the required purity on the open street, which, to say the least, is somewhat doubtful; and unfortunately for the health of the profession, here is where the larger proportion of Dental practice may be found. In addition to this it requires constant and unremitting care to avoid inhaling the breath of our patients. Of these, some may have lungs as healthy and teeth as clean, and breath as sweet as our own, (which, at best, is worthless for the purposes of respiration, immediately after it has been expired) and hence on down through every grade of nastiness, till we find some that only lacks volume of an ability and tendency to breed a pestilence. In disposing of cases of this kind we have nothing better to suggest at present, than that we hold our nose with one hand while we operate with the other, unless it be to dismiss the subject, with instructions to spend a minute and a half or two minutes every day, for a fortnight, in removing the source of the putrescent aroma that lingers around the casket of damaged pearls. Sometimes a new fangled spittoon may send up a smell, differing somewhat in character from that which might be supposed to preside in the breezes of "Araby the blast," by one who had never inhaled them, but no Dentist of good taste would long tolerate a nuisance in this quarter. The odors of the laboratory may not be at all times entirely under control, but the operating-room may, and at all times should be, kept free from all manner of exhalations, except such as may arise from the ointment of the apothecary.

The freedom and force of the circulation is intimately connected with and dependent on the functions of nutrition and respiration. It is altogether independent of the direct control of the will, though its action is accelerated during the performance of actions that are subject to its control, or during any mental excitement the arterial action is accelerated to a corresponding degree. Where there is a discord or a departure from harmonious co-operation or corresponding activity between the arterial and muscular or mental system there is a derangement that approximates disease. Medical men make the harmony between the activity of the circulation and that of the voluntary organs a test of the standard of health, and perhaps there is no single indication that may be more safely relied on. There are one or two outside influences that might be noticed in this connection. First, the temperature of the circulation must be kept at about 98°, for a very few degrees above this is termed fever heat, and is accompanied by a rapid dispersion of the material elements of the system and diminution of the vital force. In order to enable the vital organs to perform their functions regularly, it is important that the temperature be maintained at a regular standard. To meet this requirement there is an amount of warmth produced in the system proportionate to the amount of food consumed and of force expended. If an excess of heat is produced, it is thrown off by the evaporation of perspiration from the surface, and thus a regular and healthy temperature is constantly maintained. A careful observance of this law becomes especially important to the Dentist. Being confined chiefly in our occupations, he has not that constitutional vigor and tone to rely on that of right pertains to the plowman and wood-chopper, being a large part of the time deprived of the direct rays of the sun (which is doubtless the chief, if not the exclusive, source of vital energy.) Those who devote themselves to the practice of Dentistry place themselves at a great disadvantage in point of health, as compared with many other occupations, and this, together with the fact that a far less than average amount of muscular energy is expended in this than most other avocations, renders a large share of hygienic knowledge an indispensable element in a complete Dental education.

There is another external influence that may interfere with the circulation, viz: pressure. It is necessary that the arterial current be maintained without interruption or obstruction to the uttermost part and to every part of the system. If we wear our clothing too tight, and especially on our feet, we are likely to seriously injure our health, more especially as we as Dentists, exercise our feet less than in most other occupations, and idleness or disuse in any organ tends to weaken it by diverting the nervous stimulus and nutritive current of the circulation to more active members. To guard against danger from this quarter, I would suggest that every Dentist take an hour's walk once or twice each day, with little regard to the state of the weather, with strict care to guard the feet against undue pressure, and to keep them always clean, remembering that disease once established in the system, though it first find a lodgment in the utmost extremities, invariably manifests a tendency to advance toward the citadel of life.

Besides these three fundamental elements in our material organization, there is another that should not be overlooked, viz: the nervous system, the office of which is to unite the different parts of the system in one harmonious whole, and to bring us also in connection with the other portions of the universe. This, like the arterial system, is placed above and beyond the direct control of the will. Complex and incomprehensible as the more material elements of the organism may be, this is more complex, comprehensive and incomprehensible still, notwithstanding it is the medium of communication between all other parts of the system; though it guards the integrity and safety of the entire organism with the most untiring and vigilant assiduity; directs every movement and receives the impress of every emotion; is the exclusive instrument of our internal existence and our connection with the external world. Still this, like all other departments of humanity, is liable to fall a victim to, or rather become the primary seat of disease. Its functions may be depraved and perverted, or interrupted, and its structure deranged or disorganized, and whether there be a solar plexus, as some theologic anatomists propose to teach, that serves as a local habitation to the immortal spirit, there can be no question among Dentists as to the fact that there is such an affection as neuralgia, &c., or among candid, thoughtful observers, that the moral sentiments, the social affections, and the individual instincts are liable to become depraved and perverted, and this abnormal condition is not likely to long remain confined to the department where it originated, but one after another of the members are involved till the disease becomes general and at length universal. Whether the derangement originate in the osseous, muscular, alimentary or nervous systems, in the cellular, membranous or cuticular tissues, if the cause that originally wrought the mischief remains active, this sentient element transmits the diseased action or influence throughout the entire system, till the whole organism from the crown to the sole is involved.

It might be supposed that any one writing on this topic would urge the claims of some one of the numerous systems of medication that are set forth in the various medical schools of the present day, or some of the transcendently superlative nostrums that so confidently promise long life to the dying and blooming health to the hopelessly diseased, and sustain the verity of their declaration by such an ominous cloud of witnesses. This I am not prepared to do. The whole need not a physician, and the sick are scarcely competent to assume the responsibility of guiding their own steps back to the paths of life and health. It is generally easier to follow a plain, straight track, while fairly in it, than to find the way back to it after wandering off into impassable swamps and impenetrable thickets. The medical schools are chiefly based on the plan of letting the people get sick in their own way, and then curing them the best way we can.

